# Acute hemichorea in a newly diagnosed type II diabetes patient: a diagnostic challenge in resource-limited setting: a case report

**DOI:** 10.1186/s13104-016-2228-7

**Published:** 2016-08-22

**Authors:** Flora Ruhangisa, Henry Stephen, Jacob Senkondo, Amos Mwasamwaja, Said Kanenda, Saleh Mbarak, Nyasatu Chamba, Kajiru Kilonzo, William Howlett, Isaack Lyaruu, Elichilia Shao

**Affiliations:** 1 Department of Internal Medicine, Kilimanjaro Christian Medical Centre, PO BOX 3010, Moshi, Tanzania; 2 Kilimanjaro Christian Medical University College, Tumaini University Makumira, PO BOX 2240, Moshi, Tanzania; 3 Kilimanjaro Christian Medical Centre, Endoscopy Unit, PO BOX 3010, Moshi, Tanzania; 4 Better Human Health Foundation, PO BOX 1348, Moshi, Tanzania; 5 Imagedoctors International, PO BOX 16341, Arusha, Tanzania

**Keywords:** Hemichorea, Diabetes mellitus type II, Resource-limited

## Abstract

**Background:**

Chorea is a rare complication of uncontrolled type II diabetes. We report for the first time in Tanzania a case of type II diabetes presenting with a hyperglycaemia-induced hemichorea.

**Case presentation:**

A 58-year-old Tanzanian chagga by tribe with a body mass index of 28 kg/m^2^ and newly diagnosed type II diabetes presented with polydipsia and involuntary movements of the right upper limb for 4 days. His plasma glucose was 549 mg/dl and glycated haemoglobin was 18.9 %. His movements were exaggerated by attempts to use his right hand. The rest of his neurological assessment was unremarkable. Other laboratory findings including calcium were within the normal range. A computed tomography scan of the brain was essentially normal except for age-related atrophy. There was no significant ketonuria on urine dipstick testing. We treated the patient’s hyperglycaemia with intravenous insulin and the dystonia disappeared within 5 days.

**Conclusion:**

Hemichorea is among the rare complications of hyperglycaemia-induced involuntary movements. Hyperglycaemia should be considered as a differential diagnosis for patients with type II diabetes mellitus presenting with hemichorea upon clinical assessment.

## Background

Movement disorders are a common phenomenon occurring in many different medical illnesses and as adverse effects of some medications. Movement disorders are grouped into four major prototypic conditions; parkinsonism, non-parkinsonian tremor, chorea and dystonia [1, 2]. Chorea is a rare complication of non-ketotic hyperglycaemia published globally since 1985 [3]. Increasingly, literature describes different cases of C-H-BG (chorea-hyperglycaemia-basal ganglia) syndrome which has typical features of hyperintesity in the basal ganglia on magnetic resonance imaging (MRI). In settings such as ours, where MRI is not available, clinical assessment and blood investigations are most useful [4]. Most C-H-BG cases have been reported from developed countries with very few from resource limited settings and none from Tanzania [5, 6]. Diagnosing chorea associated with hyperglycaemia in Sub-Saharan Africa (SSA) can be challenging because of the wide variety of possible infectious causes; malaria, meningitis or late consequences of HIV infection such as tuberculoma and toxoplasmosis. Other non-infectious differential diagnoses include stroke, epilepsy, thyrotoxicosis and other space occupying lesions (SOL) [5].

## Case presentation

**Fig. 1:**
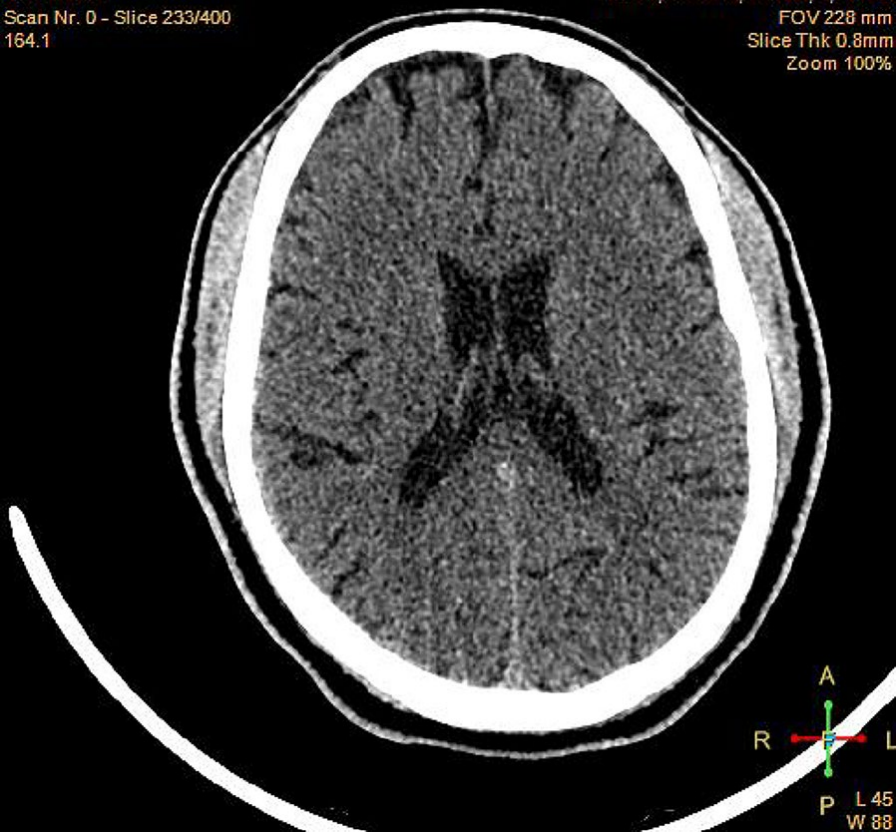
Shows normal CT-scan of the patient presented with right upper limb hemichorea due to hyperglycemia in type D.M patient in Moshi Tanzania

A 58 year old Tanzanian man, chagga by tribe with a BMI of 28 kg/m^2^ and newly diagnosed with type II diabetes mellitus was admitted to the internal medicine department of Kilimanjaro Christian Medical Centre (KCMC), a tertiary referral hospital in Northern Tanzania. His presenting complaint was polydipsia and unilateral abnormal movements of the right upper limb for four days. At presentation we observed choreiform movements of his right wrist, followed by the whole upper limb and continuing for one minute at a time. These movements were slow and repetative in a stereotypic manner, and continued intermittently, even during sleep. The choreiform movements started with a stretching of his right wrist into an abnormal spastic posture. The involuntary movements were exaggerated by attempting to perform any active movements such as attempting to shake hands with a doctor. These patterns of movement were misdiagnosed as epilepsy, stroke, cryptococcal meningitis and a SOL in the primary health centre—where there is no access to CT imaging or electroencephalogram (EEG) -prior to referral to our tertiary hospital. He was found to be hyperglycaemic (serum glucose 549 mg/dl) and to have a raised gylcated haemoglobin (HbA1c 18.9 %). His physiological observations were normal on admission, with a blood pressure of 130/70 mmHg, a pulse rate of 76 beats per minute, an axillary temperature of 36.4 °C, a respiratory rate of 19 breaths/minute and an oxygen saturation of 98 % on room air. The urine dipstick findings were glucose 4+, ketone-trace, protein-negative, pH 5 and leucocytes were trace. Serological testing for HIV, hepatitis BsAg, cryptococcus antigen and toxoplasma immunoglobulin was all negative. A chest radiograph excluded pulmonary tuberculosis and pneumonia. The patient’s full blood picture, lipid profile and biochemistry were all within normal range. A CT scan of the brain was done for exclusion of malignancy, intracerebral haemorrhage or ischemia, and was found to be grossly normal (Fig. 1). Epilepsy was excluded by a normal EEG. Unfortunately, in this setting MRI is not available but may have been helpful in confirming the diagnosis in this case.

The patient was managed with regular intravenous insulin and the frequency of choreiform movements started to decrease on the second day (serum blood glucose 270 mg/dl). On the fourth day of his admission, he was initiated on oral hypoglycaemic medications (metformin and glibenclamide). On the fifth day in hospital his serum blood glucose reduced further (129.6 mg/dl) and no more chorea was observed. On the sixth day, when his blood glucose levels were fully controlled he was discharged with oral hypoglycaemics, and his involuntary movements had resolved. On the day of discharge the patient was very happy saying ‘*I was very depressed to be told in the primary health care centre that I have epilepsy while in our family there is no one with such an illness. But after a CT*-*scan and EEG at this referral hospital and treatment it was confirmed that it wasn’t epilepsy rather hyperglycaemia*’.

## Discussion

Our case is particularly interesting because on the sixth day of admission the involuntary movements had disappeared completely. The duration of choreiform movements were very short when compared to other studies which report that chorea due to non-ketotic hyperglycaemia usually resolve within weeks to months of controlling the blood glucose [6]. This case was thought to be C-H-BG syndrome but the serum blood glucose at presentation was high, going against this diagnosis [7–9]. The serum glucose levels in our case were very high, and it is likely that the patient’s brain cells had been exposed to high levels of glucose for a long time, given the uncontrolled nature of the patient’s type II diabetes. High serum glucose is accompanied by depressed levels of consciousness and cognitive functioning. Some hypotheses state that gamma-amino butyric acid (GABA) starvation, disinhibition of dopaminergic neurons, local microhemorrhage and brain oedema may cause choreiform movements [10]. Recent imaging studies have revealed reduced cerebral glucose metabolism on positron emission computed tomography (PET) scans with hyper perfusion in the affected basal ganglia [11], important for movement’s coordination and control. Diagnostic modalities for metabolic and infectious diseases in resource-limited settings are a big challenge [12]. Some infections agents commonly found in SSA, such as mycoplasma pneumoniae, streptococcus viridians, herpes simplex and cryptococcus neoformans may also cause dystonia and chorea [13, 14]. This current case was first misdiagnosed in the primary health care centre as a form of epilepsy, stroke or SOL. It was not until this patient was seen at a tertiary level referral hospital, where CT imaging, EEG and other investigations are available that other differential diagnoses were ruled out. CT imaging among patients with chorea due to hyperglycaemia has been reported to be normal in previous studies. Though MRI was not done in our case, in other studies it has shown hyperintesity in the left basal ganglion [15]. We believe, many cases are misdiagnosed and given neuroleptic and anti-epileptic drugs unnecessarily, which can cause significant morbidity and even mortality [16]. Therefore we urge our fellow clinicians to consider the possibility of chorea as one of the rare complications of hyperglycaemia in resource-limited settings in order to reduce frequency of misdiagnosis.

## Conclusion

For the first time in Tanzania, we report a rare case of acute hemichorea in a newly diagnosed uncontrolled type II diabetic patient. From the CT imaging there was no evidence of a structural brain lesion seen. It is crucial for health care workers to consider diabetes mellitus in patients with involuntary choreiform movements.

## Abbreviations

BMI: body mass index
C-H-BG: chorea-hyperglycaemia-basal ganglia
CT: computed tomography
EEG: electro-encephalogram
GABA: gamma amino butyric acid
KCMC: Kilimanjaro Christian Medical Centre
MRI: magnetic resonance imaging
PET: positron emission computed tomography
SOL: space occupying lesion
SSA: Sub-Saharan Africa
